# The Potential of Macroporous Silica—Nanocrystalline Cellulose Combination for Formulating Dry Emulsion Systems with Improved Flow Properties: A DoE Study

**DOI:** 10.3390/pharmaceutics13081177

**Published:** 2021-07-30

**Authors:** Mitja Pohlen, Luka Pirker, Rok Dreu

**Affiliations:** 1 Faculty of Pharmacy, University of Ljubljana, Aškerčeva Cesta 7, SI-1000 Ljubljana, Slovenia; mitja.pohlen@ffa.uni-lj.si; 2 Jožef Stefan Institute, Jamova Cesta 39, SI-1000 Ljubljana, Slovenia; luka.pirker@ijs.si

**Keywords:** simvastatin, dry emulsion, lipid-based drug delivery systems, nanocrystalline cellulose, macroporous silica, spray drying, flowability, DoE

## Abstract

The objective of this study was to explore the possible use of a new combination of two excipients, i.e., nanocrystalline cellulose (NCC) and macroporous silica (MS), as matrix materials for the compounding of dry emulsion systems and the effects these two excipients have on the characteristics of dry emulsion powders produced by the spray drying process. A previously developed liquid O/W nanoemulsion, comprised of simvastatin, 1-oleoyl-rac-glycerol, Miglyol 812 and Tween 20, was employed. In order to comprehend the effects that these two matrix formers have on the spray drying process and on dry emulsion powder characteristics, alone and in combination, a DoE (Design of Experiment) approach was used. The physicochemical properties of dry emulsion samples were characterised by atomic force microscopy, scanning electron microscopy, mercury intrusion porosimetry, energy-dispersive X-ray spectroscopy and laser diffraction analysis. Additionally, total release and dissolution experiments were performed to assess drug release from multiple formulations. It was found that the macroporous silica matrix drastically improved flow properties of dry emulsion powders; however, it partially trapped the oil—drug mixture inside the pores and hindered complete release. NCC showed its potential to reduce oil entrapment in MS, but because of its rod-shaped particles deposited on the MS surface, powder flowability was deteriorated.

## 1. Introduction

The oral route of administration still remains the preferred way of administering drugs, due to patient convenience, flexibility in regimen and low production costs of this type of formulation [1]. In order for a drug to be absorbed after oral administration, it has to first dissolve in the medium of the gastrointestinal tract (GIT) [2]. It is known that, in recent decades, poorly water-soluble compounds have become prevalent in drug discovery due to new drug discovery methods, such as high-throughput screening and combinatorial chemistry [3,4]. Current predictions suggest that over 70% of newly developed drugs are hydrophobic in nature and exhibit poor aqueous solubility [5]. If a drug shows low solubility, but high permeability, and thus falls in class II of the Biopharmaceutics Classification System (BCS), formulation design and technological approaches can drastically enhance its bioavailability [3]. Within the BCS II class, drugs that are described as ‘grease ball’ type drugs mostly benefit from formulating into lipid-based drug delivery systems (LBDDS), as these often offer drug solubilisation for highly lipophilic drugs [6]. The additional benefits of LBDDS can include the drug being in a dissolved state, the facilitation of releasing the endogenous solubilisers in order to avoid drug recrystallisation in GIT and the avoidance of the first pass metabolism by exploiting the lymphatic pathway of absorption [6]. There are several different types of LBDDS systems, including lipid solutions, self-emulsifying drug delivery systems and micellar systems [7]. Based on the lipophilicity/hydrophilicity of selected ingredients, Pouton et. al. proposed a lipid formulation classification system (LFCS) containing five different types of lipid formulations, where type I consists of predominantly lipid excipients, i.e., pure oils (triglycerides or mixed mono and diglycerides) and type IV contains mostly hydrophilic excipients, such as hydrophilic surfactants and co-solvents, where the other classes are in between these two groups [3,8].

The marketed oral LBDDS are typically found in liquid form and are filled in soft or hard capsules [9]. However, liquid LBDDS have certain disadvantages, which have limited their commercial success. The main disadvantages include lower stability (risk of drug crystallisation and oxidation), potential drug leakage and manufacturing and product distribution process issues. These drawbacks can be overcome by transforming liquid LBDDS into solid ones. Apart from offering improved drug stability and lower manufacturing costs, solid LBDDS offer improved safety, as lower surfactant amounts can be used and the design of the controlled drug release is enabled [10,11]. Different manufacturing techniques have been employed for the solidification of LBDDS, from simple physical adsorption to solid carriers to more cost and energy demanding techniques, such as freeze drying and spray drying [10]. Among solid LBDDS, dry emulsions have proved to be a viable option for drug dissolution enhancement and the potential lymphatic pathway of absorption, and consequently for the drug bioavailability improvement [12,13,14]. Dry emulsions are solid LBDDS, where the outer aqueous phase of a liquid oil in a water (O/W) emulsion is replaced with a solid matrix by means of spray drying, freeze drying or pellet fluid bed coating [12,15,16]. Matrix materials are mostly sugars, proteins, cellulose derivatives and inorganic materials [12,17,18,19,20].

Cellulose is one of the most occurring natural biopolymers on Earth. Due to its broad availability, versatility, biodegradability and biocompatibility, it is widely employed in many industries [21,22]. As cellulose can be modified to obtain products with different characteristics; there is a broad array of cellulose derivatives that have been successfully adopted by the pharmaceutical industry for various applications. These polymers are hydroxypropyl methylcellulose, hydroxypropyl cellulose, methyl cellulose, ethyl cellulose, carboxymethyl cellulose, microcrystalline cellulose, etc. [23,24,25,26]. In recent years, nanocrystalline cellulose (NCC), a relatively novel cellulose product, has caught much attention in the field of biomedical sciences, because of its very high surface to volume ratio, stiffness and, depending on the production method, very different surface characteristics [27]. NCC is a material composed of rod-shaped particles with a diameter between 5–70 nm and a length of up to several micrometers. The production of NCC involves two steps; isolation of cellulosic fibres and hydrolysis, which removes the amorphous regions of the cellulose polymer [28]. Since NCC showed no toxicity to living cells, one of the most promising research areas has been focused on drug delivery. Until now, NCC has been successfully studied as a lipophilic drug delivery system agent, a thermal/pH-sensitive hydrogel former, a matrix material for long-lasting sustained drug delivery and as a disintegrant [27,29,30,31,32,33,34]. Additionally, NCC has showed great potential as a particulate stabiliser in Pickering emulsions, due to its amphiphilicity [35,36,37].

Porous silicas represent solid matrices in which rather large amounts of materials can be encapsulated [38]. Depending on their pore size range, porous silica materials can be divided in three groups: micro-, meso- and macroporous silicas [39]. Due to their high specific surface area and biocompatibility, silica materials are usually the material of choice for inorganic-based controlled drug delivery systems [38,40,41]. The use of porous silicas as materials for drug delivery ranges from amorphous solid dispersions and liquid–solid drug delivery systems to mesoporous silica nanoparticles [42,43,44,45,46].

The aim of this study is to demonstrate the feasibility of developing a dry emulsion drug delivery platform comprising a model lipophilic drug, simvastatin, in a form of a spray dried powder with enhanced drug dissolution and improved flow properties, when compared to a previously developed dry emulsion system comprising the same processing technique. For this purpose, a new combination of two matrix materials for dry emulsions, i.e., macroporous silica (MS) and NCC, has been employed. In order to thoroughly study the impact of NCC and MS, in combination and individually, on the process and powder characteristics, a Design of Experiment approach (specifically, a response surface design with three formulation variables) has been used. Powder flow, one of the main product quality attributes in our study, has been studied by a classic pharmacopoeian method, i.e., Carr index, and by an advanced automatic rotating drum technique. Other DoE-studied response quality attributes were particle size, oil droplet size after reconstitution, drug encapsulation, the percent of released drug and the process yield.

## 2. Materials and Methods

### 2.1. Materials

Simvastatin ([(1S,3R,7S,8S,8aR)-8-[2-[(2R,4R)-4-hydroxy-6-oxooxan-2-yl]ethyl]-3,7-dimethyl-1,2,3,7,8,8a-hexahydronaphthalen-1-yl] 2,2-dimethylbutanoate) was kindly donated by Krka d.d., Novo Mesto, Slovenia. 1-oleoyl-rac-glycerol (1OG) (technical grade ∼40% (TLC)), and Tween^®^ 20 (polyethylene glycol sorbitan monolaurate) was purchased from Merck, Darmstadt, Germany. Hydrophilic sodium salt of sulphated nanocrystalline cellulose (CelluForce NCV100) was purchased from CelluForce, Montreal, QC, Canada. Macroporous silica (SP53D-12096) was donated by Grace, Worms, Germany. Pearlitol SD 200 (mannitol) was purchased from Roquette, Lestrem, France. Pharmcoat 603 (Hydroxypropyl Methycellulose—substitition type 2910 (USP), 3cP) and Miglyol^®^ 812 (M812) were supplied by ShinEtsu, Tokyo, Japan and Sasol, Hamburg, Germany, respectively. Solvents for U(H)PLC analysis were of HPLC (high performance liquid chromatography) grade. All other reagents used were of analytical grade. Water for the UPLC analysis was purified with a Milli-Q system with a 0.22 µm Millipak 40 filter (Millipore, Tullagreen, Ireland).

### 2.2. Methods

#### 2.2.1. Preparation of Liquid Emulsions

The liquid emulsion composition (selection of components) and preparation were based on results from a previous article and modified in order to include insoluble matrix formers [12]. Briefly, 1OG was heated up to 40 °C in order to obtain a clear liquid and mixed with M812 in the ratio of 9:1. Tween^®^ 20 was added to the prepared mixture up to 0.5% (m/m) and then mixed. After the oil–surfactant solution was prepared, simvastatin in the amount of 70 mg per gram of oil–surfactant solution, was added, dispersion heated to 37 °C and mixed with magnetic stirrer until a clear solution was obtained. The oil–surfactant mixture drug solution, referred as the oil phase, was kept at 37 °C.

The composition of the external—matrix phase and its ratio to the oil phase was varied according to the experimental design described in Section 2.3. The mannitol solution was heated to 37 °C and the oil mixture was added during stirring with propeller agitator at 640 rpm. The resulting pre-emulsion was firstly homogenised using a high shear, rotor–stator, homogeniser (Ultra-Turrax^®^ T25, IKA-Works, Staufen, Germany) for 5 min at 8000 rpm and 3 min at 12,000 rpm. HPMC (hydroxypropyl methylcellulose) was prepared as a separate solution to prevent HPMC chain degradation during high shear, rotor–stator homogenization [47,48]. The HPMC solution was added to pre-emulsion during mixing with propeller agitator at 640 rpm for 3 min. Finally, a two-stage high pressure homogeniser (APV—2000, SPX flow technologies, Silkeborg, Denmark), with 300 bar for the first stage was used in order to obtain the emulsion. The high pressure homogenisation was repeated nine times. Depending on the experimental design, MS or NCC in powder, alone or in combination, was added to the prepared emulsion. After the addition of the two insoluble matrix formers, the final emulsion was mixed with propeller agitator at 640 rpm for 2 more hours.

#### 2.2.2. Characterisation of Liquid Emulsions

Oil droplet size distributions after the high pressure homogenisation and after reconstitution of dry emulsion samples were measured by laser diffraction (Mastersizer S, Malvern Instruments, Ltd., Malvern, UK) using the 300 RF lens and a small volume liquid dispersion unit (at 1000 rpm) with the following parameters: 20 ± 2.5% obscuration rate and refractive index for the oil phase of 1.46. The droplet size was described by volume-based distribution parameters (d10, d50, d90 and SPAN, where SPAN is calculated as SPAN = (d90 − d10)/d50). Measurements were performed in triplicate and expressed as an average ± standard deviation (SD). In the case of reconstituted dry emulsion samples, the size of oil droplets was in the range up to 20 µm, while silica particles were well separated with size from 56 µm and above; therefore, a cut-off of 56 µm was used for evaluation of reconstituted emulsions.

#### 2.2.3. Atomic Force Microscopy (AFM)

The size distribution of NCC was obtained with a non-contact frequency-modulated atomic force microscope (NC-AFM, Omicron VT-AFM, Taunusstein, Germany) operating in ultra-high vacuum (10^−9^ mbar). The NCC was dispersed in ultra-pure water and drop casted on a freshly cleaved highly oriented pyrolytic graphite (HOPG) substrate. The sample was dried before analysis.

#### 2.2.4. Spray Drying Process

##### 2.2.4.1. Process Parameters

Spray drying processing was used (Mini spray dryer B-290, Büchi, Flawil, Switzerland) to quickly immobilise the oil droplets during drying in order to obtain droplets embedded in the matrix. A two-fluid nozzle with 1.4 mm opening diameter and a 2.20 mm cap opening diameter was used. The spray drying process parameters were as follows: drying gas flow rate of 28 m^3^/h (70% aspiration rate); product temperature was kept between 75 °C and 80 °C by adjusting the inlet temperature; spraying rate of 6 g/min; flow meter spraying air (atomization gas flow rate) was 443.5 L/h; nozzle geometry, as described in previous publication [49], was set to 0 mm, which means that the nozzle and cap were parallel, forming a narrow spray pattern. After the spraying was completed, dry emulsion powders were further dried for 3 min at the product temperature of 80 °C to further lower the moisture content in the final product. Powders were collected from the cyclone, the product collection vessel, separation flask and the drying chamber and then all fractions were combined and mixed. The aforementioned collection procedure was carried out to obtain enough products for planned analyses.

##### 2.2.4.2. Spray Drying Process Yield

The spray drying yield was calculated from the useful product mass and the loss on drying (85 °C, 15 min) moisture content of obtained powders using Equation (1). Particles greater than 800 µm were considered as waste and were eliminated before weighing by sieving through an 800 µm sieve.

(1) $$\mathrm{Process}\;\mathrm{yield}=\frac{\mathrm{mass}\;\mathrm{of}\;\mathrm{spray}\;\mathrm{dried}\;\mathrm{powder}\;\times \left(100\;\%\;-\;\mathrm{percent}\;\mathrm{of}\;\mathrm{moisture}\;\mathrm{in}\;\mathrm{the}\;\mathrm{powder}\right)}{\sum \left({\mathrm{mass}}_{i}\times \left(\left(100\;\%\;-\;\mathrm{percent}\;\mathrm{of}\;\mathrm{moisture}\;\mathrm{in}\;\mathrm{the}\;i-\mathrm{th}\;\mathrm{component}\right)/100\right)\right)}$$

where mass_i_ is the mass of i-th dispersion component that was sprayed during the drying experiment.

#### 2.2.5. Characterisation of Dry Emulsion Powders

##### 2.2.5.1. Drug Content

500 mg of dry emulsion powders were added to 25 mL of methanol and sonicated for 20 min in order to ensure complete release of the drug into the medium. The resulting dispersion was diluted with methanol to obtain a final theoretical simvastatin concentration of approx. 10 µg/mL. Finally, the dispersion was filtered through a 0.22 µm PTFE syringe filter and assayed with UPLC method (method described in Section 2.2.5.12. U(H)PLC analysis). Results were expressed as milligrams of simvastatin per gram of product.

##### 2.2.5.2. Extent of Released Drug

To determine the extent of the released drug from dry emulsion samples, the following procedure was used. Approximately 400 mg of product was accurately weighted and placed in a conical centrifuge tube. A total of 10 mL of purified water was added and mixed for 1 min on a vortex shaker, 15 min on a horizontal shaker and again 1 min on a vortex shaker. Samples were then placed in a centrifuge (Centric 322A, Tehtnica, Železniki, Slovenia) on 500 RPM for 5 min. Mild separation conditions were used in order to avoid phase separation, but at the same time allowing insoluble matrix sedimentation. At the end of the preparative part, 1 mL of supernatant was carefully withdrawn, diluted with methanol and analysed, as described in Section 2.2.5.12. U(H)PLC analysis. The amount of released drug was expressed relative to the drug content as final percentage of released drug.

##### 2.2.5.3. Encapsulation Efficiency

Encapsulation efficiency (EE) was calculated as drug content embedded in the matrix against theoretical amount of drug in sprayed dispersion by employing Equation (2):

(2) $$\mathrm{Encapsulation}\;\mathrm{efficiency}\;\left(\mathrm{EE}\right)\;=\;\frac{\mathrm{drug}\;\mathrm{content}\;\left(\mathrm{product}\right)\times \mathrm{mass}\;\mathrm{product}}{\mathrm{mass}\;\mathrm{of}\;\mathrm{simvastatin}\;\mathrm{in}\;\mathrm{the}\;\mathrm{sprayed}\;\mathrm{liquid}\;\mathrm{emulsion}}\times 100\%$$

##### 2.2.5.4. Moisture Content

The moisture content of the dry emulsion powders was determined gravimetrically as loss on drying, employing the Büchi moisture analyser (B-302, Büchi, Flawil, Switzerland) by heating approx. 3 g of powder for 15 min at 85 °C. The moisture content was calculated using Equation (3).

(3) $$\mathrm{Moisture}\;\mathrm{content}\;\left(\%\right)=\frac{{\mathrm{sample}\;\mathrm{mass}}_{\mathrm{before}\;\mathrm{drying}}-{\mathrm{sample}\;\mathrm{mass}}_{\mathrm{after}\;\mathrm{drying}}\;}{\mathrm{dry}\;\mathrm{emulsion}\;\mathrm{powder}}\times 100\;\%$$

##### 2.2.5.5. Particle Size Analysis

Dry emulsion and pure MS particle size distributions were measured by a laser diffraction measurement (Mastersizer S, Malvern Instruments, Ltd., Malvern, UK) using the 300 F lens and a dry powder feeder unit with the following parameters: feed air pressure 3 bar; 0.5–5% obscuration rate; Fraunhofer theory setting. The particle size distribution was described by volume-based distribution parameters (d10, d50, d90 and SPAN). Measurements were undertaken in triplicate and expressed as an average ± standard deviation (SD).

##### 2.2.5.6. Scanning Electron Microscopy (SEM)

Scanning electron microscopy was used to determine the morphology of the spray dried powders. Samples to be analysed were placed on a graphite foil and examined with a 235 Supra 35VP-24-13 high-resolution scanning electron microscope (SEM) (Carl Zeiss, Oberkochen, Germany) at 1.0 kV acceleration voltage and different magnifications, using secondary electrons as a signal.

##### 2.2.5.7. Dry Emulsion Reconstitution

In order to evaluate the spray dried dry emulsion powder reconstitution, conditions resembling the in vivo situation, in terms of drug to liquid ratio, were recreated. A product sample equivalent to 40 mg of simvastatin taken with 200 mL of water was placed in 40 mL of distilled water within a conical centrifuge tube mounted on a horizontal shaker (amplitude 1 cm and a frequency of 150 RPM) for 15 min and shaken additionally for 1 min on a vortex shaker. The shaking procedure was repeated twice. Afterwards, the size distribution of the oil droplets was measured, as described under Section 2.2.2. Characterisation of liquid emulsions. In order to be able to distinguish between the insoluble matrix formers and the emulsion, measuring channels comprising sizes bigger than 56 µm were eliminated. All experiments were carried out in triplicate.

The oil droplet size distribution index (SDI) is a single value index appropriate for modelling, proposed to better represent the bimodal size distribution of droplets obtained after reconstitution. The size distribution index logic is discussed in the related article and is calculated as follows [12]:

(4) SDI=AUC(1)×MAX(1)+AUC(2)×MAX(2)

where AUC is the area under curve of the volume-based size distribution peak (expressed in volume percentage), and MAX is the maximum of the peak (expressed as size). The delimitation between the peaks used to calculate the AUC was the minimum value between the peaks. Monodisperse narrow size distributions express low SDI index values, as opposed to polydisperse distributions.

##### 2.2.5.8. Mercury Intrusion Porosimetry

The total pore volume and average pore diameter of the MS powder and spray dried dry emulsion products were determined using a mercury intrusion porosimeter (Pascal 140 and Pascal 440, Thermo Fisher Scientific, Waltham, MA, USA), with pressures from 10 kPa up to 400 MPa (corresponding to a pore diameter interval 150 μm–3.2 nm). The surface tension and the contact angle of the mercury were set to standard values of 0.485 mN/m and 130°, respectively.

##### 2.2.5.9. Energy-Dispersive X-ray Spectroscopy (EDS)

Chemical analysis was made with a FEI HeliosNanolab 650 (Thermo Fischer Scientific, Waltham, MA, USA) scanning electron microscope (SEM) using energy-dispersive X-ray spectroscopy (EDS). All the samples were coated with approximately 10 nm of carbon with the aim to prevent charging effects during electron irradiation. To determine the presence of different elements, an area approximately the size of 100 × 100 μm^2^ was selected on chosen samples.

##### 2.2.5.10. Flow Properties

Two methods were employed to measure the flow properties of powders.

The first method, Hausner ratio, is widely used to evaluate the flow properties of powders based on the difference between bulk and tapped density, which is in correlation with the powder cohesion [50,51,52]. Hausner ratio (HR) is calculated as follows:

(5) $$\mathrm{HR}=\frac{\mathrm{Vb}}{\mathrm{Vt}}$$

where Vb stands for bulk volume and Vt stands for tapped volume.

The second method employed Mercury Scientific Revolution Powder analyser (RPA) (Newtown, CT, USA) in order to assess sample flow properties under dynamic conditions. RPA analyses powder characteristics during powder avalanching. The parameters during analysis were the subsequent: drum speed: 0.3 rounds per minute; image threshold: 60; frequency of image capturing: 30 images/second; measurement time: until 150 avalanches were observed. Before the actual measurement, samples were prepared as follows: a 100 cm^3^ of sample was accurately weighted, placed in the rotating drum and then potential electrostatic charge was reduced by passing the drum through a static discharger unit. Two metrics were used for describing powder sample flow properties, i.e., avalanche angle median and surface fractal. For a deeper description of the design and operation, the reader is directed to the study of Nalluri et al. [53].

##### 2.2.5.11. Dissolution Studies

Spray dried dry emulsion products were tested for dissolution by USP II dissolution apparatus. An accurately weighted amount of product, equivalent to 20 mg of simvastatin, was introduced in 500 mL of the dissolution medium containing citrate buffer solution with a pH = 4.0 (10.05 g/L citric acid, 8.0 g/L sodium hydroxide, adjusted with hydrochloric acid). The paddles were rotated at 100 rpm and the temperature was maintained at 37 °C ± 0.5 °C. At predetermined time intervals (1, 3, 5, 10, 15, 30, 60 and 120 min) samples were withdrawn (without replacing the medium with fresh buffer) and diluted with methanol in ratio 1:3 = sample:methanol. Prior to analysis, samples were filtered through a 0.22 µm filter and analysed with U(H)PLC (described in Section 2.2.5.12. U(H)PLC analysis). All dissolution experiments were performed in triplicates.

##### 2.2.5.12. U(H)PLC Analysis

The UPLC method was previously developed and described (Pohlen et al., 2018). Simvastatin was determined by the chromatographic system Acquity UPLC (Waters Corp., Milford, MA, USA). A UV–VIS photodiode array (PDA) module equipped with a high sensitivity flow cell was used for detection. The column used was a reverse phase column Acquity UPLC BEH C18 1.7 µm; 2.1 × 100 mm (Waters Corp., USA). A gradient elution was used, containing mobile phase A (90% water, containing 0.1% orthophosphoric acid and 10% acetonitrile) and mobile phase B (98% acetonitrile and 2% water). The gradient method was the following: start at 50:50 (A:B); 0–6 min, 50:50–40:60; 6–7 min, 40:60; 7–8 min, 40:60–50:50; 8–10 min, 50:50. The flow rate was set at 0.3 mL/min and the column temperature was kept at 45 °C. The auto-sampler temperature was set at 10 °C. The injection volume was 5 µL and the run time was 10 min. Simvastatin and its acid form were detected at the wavelength of 238 nm. The retention times were 4 min and 6 min, for simvastatin hydroxyacid and simvastatin, respectively.

### 2.3. Experimental Design

First, different emulsion formulations consisting of 1-oleoyl-rac-glycerol with Miglyol^®^ 812—9:1 fixed ratio (oil), mannitol, Pharmacoat^®^ 603 (HPMC), MS, NCC and Tween^®^ 20 were tested by spray drying processing in order to set limits beyond which the formulations were not processable anymore. The ratio between the soluble matrix formers was kept constant, i.e., ratio HPMC:mannitol = 1:5.8. The set limits for different components were: oil phase with surfactant and dissolved simvastatin (70 mg/g) was varied from 27% to 40% (Tween^®^20 concentration was kept at 0.5% for all experiments), NCC from 0% to 10%, MS from 0% to 50% and the remaining percent up to 100% of non-volatile substances was completed with soluble matrix formers, i.e., HPMC and mannitol. The non-volatile substance concentration in the emulsion, without the API, was kept at 30%, as it was discovered in the previous study that this concentration was beneficial for increasing particle size [49]. For the experimental design and statistical evaluation, Minitab^®^ 17 software (Minitab Inc., State College, PA, USA) was used. Response surface design with three formulation variables was used and three repetitions were made at the central point in order to estimate the repetition error. The three independent variables were: oil phase concentration (X1), NCC concentration (X2) and MS concentration (X3). Process yield (Y1), encapsulation efficiency (Y2), amount of released drug (Y3), median particle size, d50_particles_ (Y4), mode of the droplet size distribution (SDI) (Y5), HR (Y6), avalanche angle median (Y7) and surface fractal (Y8) were taken as DoE responses for modelling. In total, 17 experiments were performed with three repetitions in the central point, as shown in Table 1. When setting the models, a stepwise elimination with α = 0.15 (except for modelling SDI, where α = 0.16 was chosen, as in this way NCC was still retained in the model) criterion was used to eliminate variables that were not significant for a given response.

## 3. Results and Discussion

### 3.1. Liquid Emulsions

The droplet size distribution of the liquid emulsions was evaluated before the addition of the insoluble matrix formers in order to be sure that the liquid emulsion preparation method gave consistent results among all experiments. The d50 of all the experiments was 0.33 ± 0.06 µm. This result confirms that the size distribution of droplets was comparable among experiments before the drying step, and thus eliminating the potential effect of the initial emulsion droplet size distribution on the final characteristics of the product.

### 3.2. NCC and MS Characterisation

**Figure 1:**
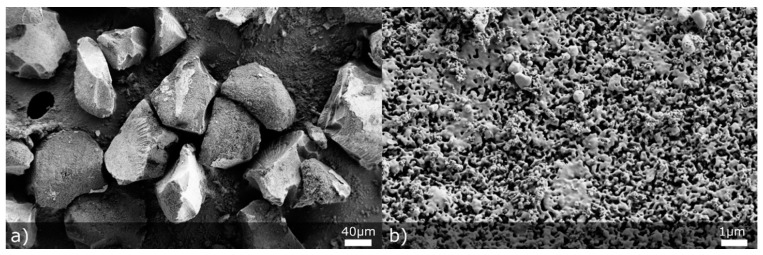
SEM pictures representing initial MS particles at (**a**) 500× and (**b**) 20,000× magnification.

MS was characterized according to particle and pore size. One of our motives in using MS particles was to increase particle size of the final spray dried product and thus improve flow properties, which is why bigger MS particles were preferred during the preliminary matrix screening phase. Initial MS particle size distribution, assessed by laser diffraction measurements using a dry dispersion cell, was the following: d10 = 119 ± 1 µm, d50 = 209 ± 1 µm and d90 = 327 ± 4 µm. As can be seen from SEM micrographs (Figure 1a), MS particles are of irregular shape with clear cut surfaces. Based on these results, it was assumed that an increased percentage of MS in the formulation would improve flow properties. With mercury porosimetry, we have also assessed the average pore size of MS, which was 489 nm. This result classifies our silica material as macroporous silica (average pore size greater than 50 nm) [39]. The reasoning for employing macroporous material (instead of mesoporous material) in our study was to allow NCC and individual oil droplets to enter MS pores and to study if NCC would improve oil phase release.

**Figure 2:**
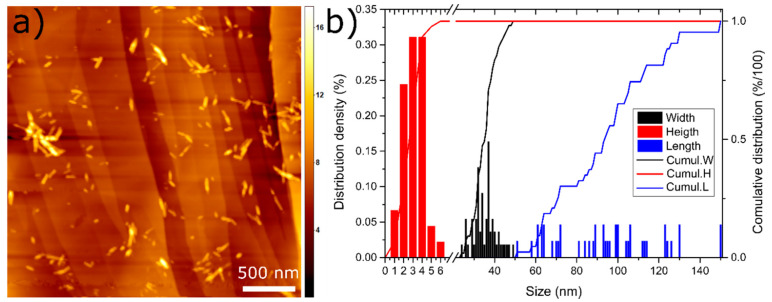
(**a**) AFM image of the nanocrystalline cellulose (NCC) on the highly oriented pyrolytic graphite (HOPG) surface. The scale bar is 500 nm, the legend on the right is in nm and shows the height of the particles; (**b**) distribution density and cumulative distribution for the width, height and length of the NCC particles.

NCC comes as a spray dried powder and is a sulphate salt of cellulose, thus having a more hydrophilic character compared to other modified NCCs [54]. Once dispersed in water, rod-shaped crystallites with 34 ± 6 nm in diameter, 94 ± 25 nm in length and 3 ± 1 nm in height are formed, as shown by Figure 2a,b.

### 3.3. Dry Emulsion Powders

#### 3.3.1. Process Yield

Process yield can be a challenge when spray drying LBDDS due to the inherent stickiness of the lipid components. An additional problem in academic research laboratories is the small scale equipment, which limits the process yield because of the narrow drying tower, leading to material being adhered to the drying tower wall [55]. The process yield in our study ranged from 65.22% to 91.99% with an average value of 85.69%. The model ((R^2^ = 0.6976, R^2^(adj) = 0.5162, R^2^(pred) = 0) extracted from the process yield data is described by Equation (6):

(6) $$\mathrm{Process}\;\mathrm{yield}\;\left(\%\right)=-17.8+612\times X1+237\times X2+67\times X3-869\times X1^{2}-833\times X1\times X2-198.7\times X1\times X3$$

The model has shown that the process yield increases by decreasing the oil phase content and increasing the content of matrix formers (both NCC and MS). This can be expected, as a higher amount of oil phase will lead to more oil on the particle’s surface, resulting in particles sticking to the surface of the drying tower. On the other side, increasing the amount of insoluble matrix formers decreases the stickiness of the spray dried particles in a more efficient way than an increase in soluble matrix formers, leading to an increased process yield.

#### 3.3.2. Drug Content

**Figure 3:**
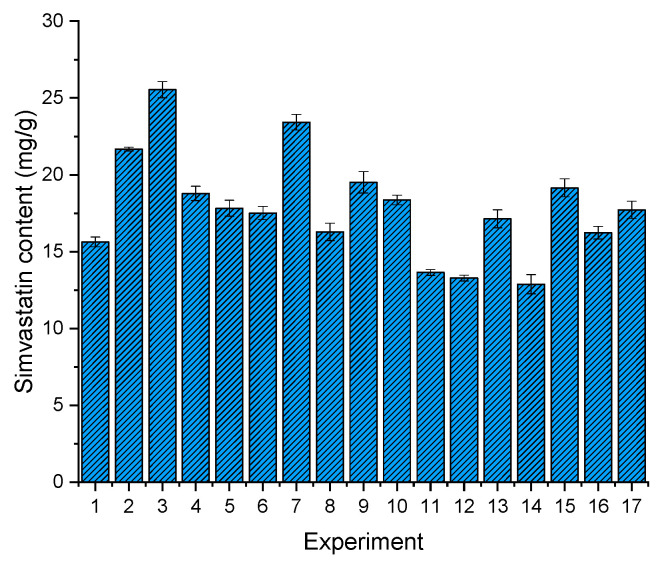
Drug content across 17 experiments, determined by UPLC analysis.

When transforming liquid LBDDS into solid LBDDS, the drug content is one of the biggest issues, as the addition of solid components to the formulation reduces the concentration of the active ingredient in the final product, which can already be impaired by the solubility in the lipid media [56]. Since raising the amount of matrix formers in general improves the flow properties of solidified LBDDS, a compromise should be taken between the drug content and processability/flowability of the obtained powders. Drug contents among 17 formulations are shown in Figure 3. The lowest drug content of 12.88 ± 0.62 mg/g was achieved with F14, while the highest drug content of 25.54 ± 0.52 mg/g was achieved with formulation F3. The average drug loading of 17.91 mg/g could seem low at the beginning, especially when we look at the normal doses of our model compound, which range from 20 mg to 80 mg. However, if we envisage an improvement in drug absorption, and thus drug bioavailability due to the lipid based formulation, the modest loading can be accepted, as the pharmacological effect of the 20 mg simvastatin usual dose could be reached with an acceptable dosage unit mass [49,57]. Dry emulsions have proved many times that they possess the ability to significantly improve bioavailability [13,58].

#### 3.3.3. Encapsulation Efficiency

Encapsulation efficiency is very important when transforming liquid emulsions into solid dry emulsions, as a considerable portion of the active ingredient–oil mixture can be potentially lost during the spray drying process, thus lowering the amount of the encapsulated active ingredient and increasing production costs. This is why encapsulation efficiency was analysed and modelled. The obtained model has reasonably high coefficients of determination (R^2^ = 0.7866, R^2^(adj) = 0.7154, R^2^(pred) = 0.4849) and has the following form (Equation (7)):

(7) $$\mathrm{EE}\;\left(\%\right)=-144.5+1241\times X1+95.7\times X3-1667\times X1^{2}-390\times X1\times X3\;$$

**Figure 4:**
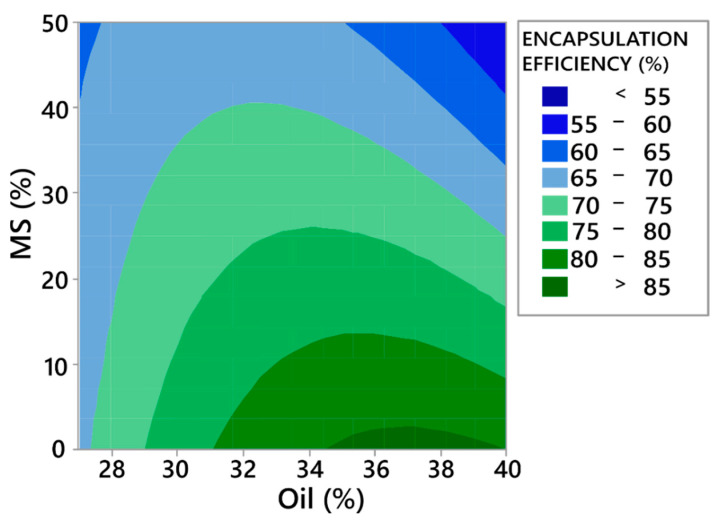
Contour plot showing the effect of oil and MS percentage on encapsulation efficiency.

The model reveals that only concentrations of the oil phase and MS affect the encapsulation efficiency. From the contour plot on Figure 4, it can be observed that increasing the MS concentration lowers encapsulation efficiency. This can be explained by the fact that a higher MS concentration lowers the concentration of soluble matrix formers in the formulation (mannitol and HPMC), which offer better encapsulation than the sole inorganic carrier (F17). This is because during the drying process, or, more precisely, after the atomisation phase, there is a very limited time for the oil phase to completely enter the MS pores. The oil phase left on the surface of particles is thus susceptible to transfer to the surface of the equipment, lowering the encapsulation efficiency. If soluble matrix formers are present in the formulation, they tend to efficiently encapsulate the oil phase, even on the surface of MS particles. On the contrary, increasing the concentration of oil (in the lower MS concentration range) increases the encapsulation efficiency, which was also found in the study by Hansen et al. [59]. This observation can again be explained by the higher presence of the soluble matrix formers in formulations with high oil phase and low MS concentrations, as the percent of the solid phase was fixed to 30% for all formulations. 

#### 3.3.4. Amount of Released Drug

Porous inorganic materials, such as Aerosil^®^, Sylysia^®^, Neusilin^®^ etc. are very efficient in loading LBDDS due to their high porosity; however, many studies have shown that in vivo and in vitro performance can be noticeably reduced because of incomplete desorption of the liquid lipid part of the LBDDS from the inorganic carrier [41,60,61]. It has been found that this happens especially where the average pore size is large and where there is a wider pore size distribution [61]. This is the reason why the amount of drug released from the formulations was evaluated and modelled. The model we obtained fitted the release data extremely well, which is shown by high coefficients of determination (R^2^ = 0.9608, R^2^(adj) = 0.9430, R^2^(pred) = 0.8983). The following equation describes the model:

(8) $$\mathrm{Released}\;\mathrm{simvastatin}\left(\%\right)=361.1-1574\times X1+49.2\times X2-155.9\times X1+2275\times X1^{2}+491\times X2\times X3$$

**Figure 5:**
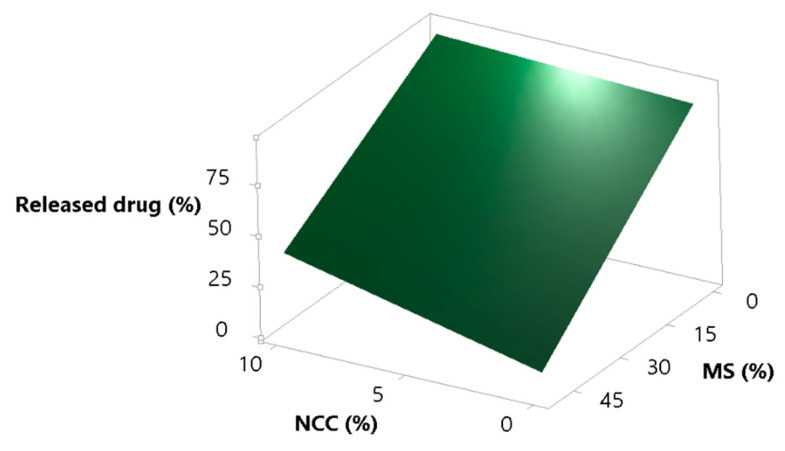
Surface plot for the response percent of released drug in the supernatant; effect of NCC and MS concentration.

It was expected that the increase in MS in the formulation would decrease the percent of released simvastatin, and that NCC would at least partially cover lipid droplets and cover/fill the MS pores and act as a desorption enhancer. These assumptions are correct, as can be seen from Figure 5. Increasing the concentration of MS in the formulation strongly decreases the percentage of released simvastatin, but this phenomena is somewhat opposed by NCC, which, as assumed, eases lipid desorption [62,63].

#### 3.3.5. Particle Size and Morphology

**Figure 6:**
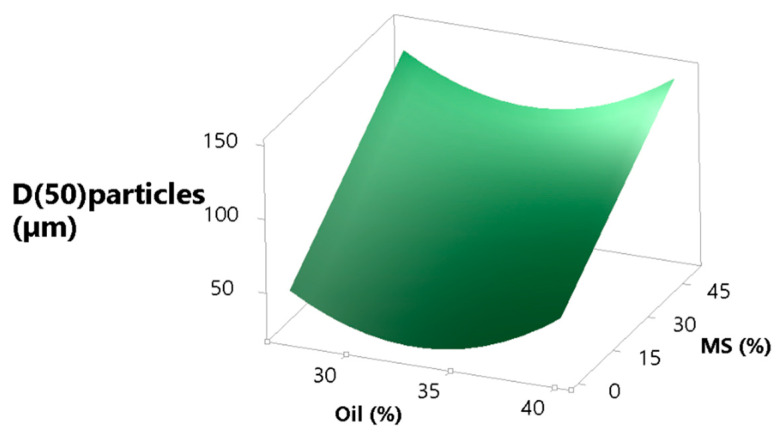
MS and oil concentration effect on the size of spray dried particles, expressed as d(50).

Beside other particle characteristics, e.g., particle morphology, moisture, etc., particle size is generally regarded as one of the most important parameters with respect to flowability, which is, apart from the high particulate density, one of the reasons why MS was added to the formulation [64,65,66]. In our study, MS should partially act as a seed particle to be coated with the dry emulsion and partially as a porous particle in which dry emulsion should be loaded. As expected, increasing the MS concentration leads to an increased particle size, as is shown in the surface plot (Figure 6). The function (Equation (9)) describing the effect of oil on the particle size (expressed as d50) has a parabolic shape with the minimum in the middle. We can speculate that a high oil concentration leads to more oil on the particle surface after drying, which acts as a sort of binder, weakly sticking particles together. On the other end of the quadratic curve of oil (lower concentration of oil) we have either a more water-soluble matrix, which could also have the same binding effect as oil, but with stronger bonds compared to oil, or more MS, which alone increases particle size. The equation describing the effect that oil and MS have on particle size is the following:

(9) $$\begin{array}{c} D\left(50\right)\mathrm{particle}=791-4657\times X1+175.3\times X3+7070\times X1^{2}\\ \left(R^{2}=0.6468,{\;R}^{2}\left(\mathrm{adj}\right)=0.5653,{\;R}^{2}\left(\mathrm{pred}\right)=0.4040\right) \end{array}$$

**Figure 7:**
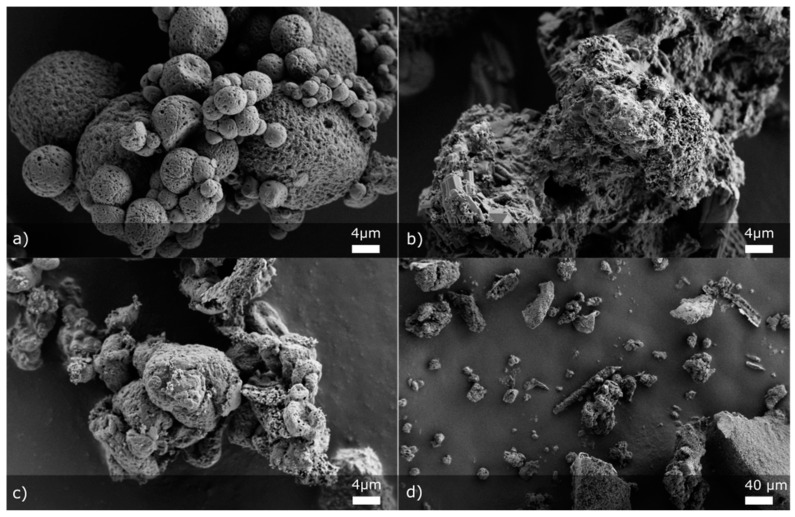
SEM micrographs of four experiments with two different magnifications: (**a**) F1, (**b**) F14, (**c**) F17 (all three 5000× magnification) and (**d**) F14 (500× magnification).

With SEM pictures of selected samples, the morphology of the particles was studied. For that, we chose three formulations: F1 without insoluble matrix formers (Figure 7a), F14 where only MS as an insoluble matrix former at the highest concentration was present (without NCC) (Figure 7b,d) and F17 with both insoluble matrix formers at the highest concentration (Figure 7c). From Figure 7a, we can see that in the case where we have only soluble matrix formers, small, mostly round-shaped, aggregated particles are produced. Pores filled with oil are clearly seen on the surface of formed matrix. Looking at Figure 7b, one can see an MS particle surface covered with soluble matrix formers. The product of the same experiment (F14) is shown in Figure 7d, but at a smaller magnification, where MS particles after drying are depicted more clearly. Figure 7c shows a part of the product from the experiment F17. We cannot clearly see any MS particles in this figure, which shows that throughout the sample there should be some non-homogeneity. However, we can note the difference between particles that have only soluble matrix formers (Figure 7a) and particles with added NCC in the formulation (Figure 7c). We can observe that the addition of NCC produces particles with lower sphericity, showing a potential disrupting effect of NCC on the formation of the isometric particle shape during drying.

#### 3.3.6. Spatial Distribution of Components

**Figure 8:**
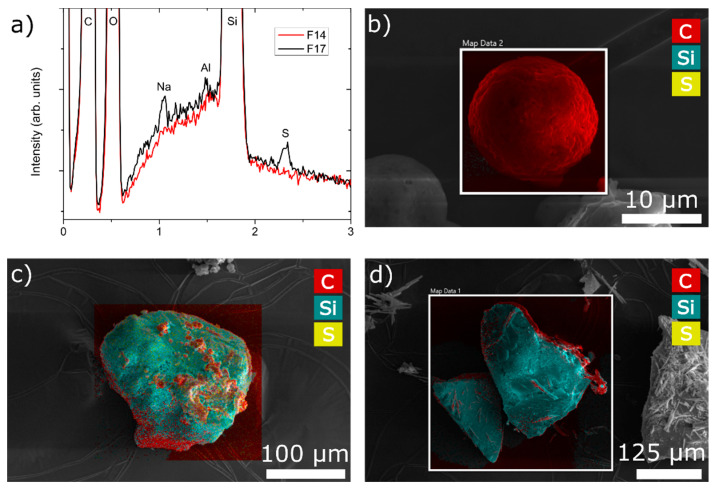
(**a**) Summed EDS spectra of mapping performed on samples F14 and F17; EDS mapping performed on three samples: (**b**) F1; (**c**) F17; (**d**) F14, where red colour represents carbon atoms (HPMC, mannitol), yellow colour represents sulphur atoms (NCC) and blue colour represents silicium atoms (silica).

EDS was employed to determine the presence and spatial distribution of chemical elements and, hence, excipients containing them. Three samples were analysed; F1, F14 and F17. F14 is composed of the maximum amount of MS, whereas F17 also has the maximum amount of NCC. F1 does not have either MS or NCC. As can be seen from Figure 8c,d, both F14 and F17 are rich in Si, which is the main constituent of MS. The main difference between the two samples (Figure 8a) can be found in the presence of sulphur (S) and sodium (Na), which is a part of NCC (cellulose sulphate sodium salt). A map of the sample particle F1 is depicted all in red (Figure 8b), which is consistent with its composition: rich in carbon. Looking at Figure 8c, one can see the spatial distribution of excipients in the dry emulsion powder, where all formulation components were used. We can see that MS particles form the core on (and within) which other components of the formulation are deposited. NCC is evenly distributed, whereas soluble matrix formers are found in clusters. In the case of the F14 particle surface map (Figure 8d), one can see a blue silica core with some defined needle-like structure rendition in red, which could be ascribed to the crystallization of mannitol on the particle surface.

#### 3.3.7. Reconstitution Ability of Dry Emulsion System

As the size of lipid droplets in the gastrointestinal space is an important factor when it comes to drug bioavailability enhancement and a more predictable absorption with LBDDS, the reconstitution ability of dry emulsions was assessed by looking at the mode of the droplet size distribution after reconstitution [56,67]. The model describing SDI (R^2^ = 0.6819, R^2^(adj) = 0.4910, R^2^(pred) = 0.0248) yielded the following equation:

(10) $$\mathrm{SDI}=0.523+1.12\times X1-0.98\times X2+2.19\times X3-2.58\times X3^{2}-5.53\times X1\times X3+6.3\times X2\times X3$$

From Equation (10), we can extract that high oil concentration is desired for obtaining low SDI values. We hypothesised that NCC would act as a Pickering emulsion stabiliser [68], but this was not the case. In the concentrations we used, the function of NCC exhibits a negative effect on the droplet size distribution. The detrimental effect of NCC on SDI can be explained by the morphology of particles (Figure 7c). It seems that when NCC is added in excess, at least part of NCC encapsulate the oil phase on its own and deforms the oily droplet phase during the drying stage. The same is not true for MS particles, whose high concentration significantly improves the redispersibility (low SDI values), which can be due to the possible mechanism of oil droplets being individually entrapped in the pores of the macroporous matrix, thus preventing oil coalescence.

#### 3.3.8. Porosity

**Figure 9:**
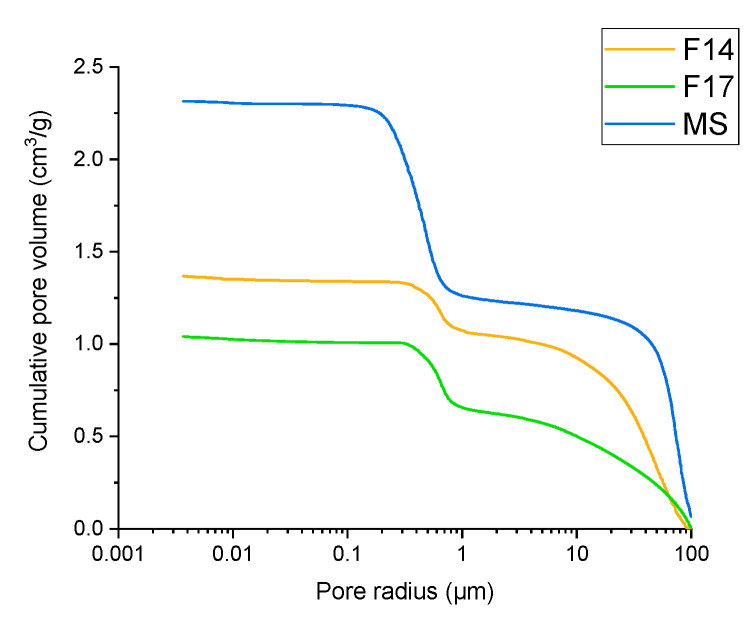
Dependency of cumulative pore volume on pore size as determined by mercury intrusion porosimetry for pure MS (blue line), experiment F14 (orange line) and experiment F17 (green line).

In addition to initial MS particles, samples of two spray dried products were analysed for porosity with mercury intrusion porosimetry, i.e., F14 and F17. Pure MS showed a total pore-specific volume of 1.051 cm^3^/g, compared to the total pore volume of 0.292 cm^3^/g and 0.383 cm^3^/g, for experiment F14 and F17, respectively. Looking at the average pore diameter derived from the cumulative pore volume as a function of pore size as determined by mercury intrusion (Figure 9), we can see that MS had an average pore diameter of 489.1 nm, compared to smaller pore diameters of 340.6 nm for experiment F14 and 250.1 nm for experiment F17. Two conclusions can be extracted from these results. Firstly, it is clear that, independently of the matrix material (NCC or soluble matrix formers), part of the MS pores are filled with matrix/oil material, which is shown as a decreased total pore-specific volume in the samples F14 and F17. Additionally, we can see that experiment F14, which, in comparison to experiment F17, also contains soluble matrix formers, exhibits a lower pore volume, while F17 with added NCC particles demonstrates a smaller average pore diameter. This shows that either soluble matrix formers or NCC particles, apart from the oil phase, fills the pores of MS matrices efficiently.

Only the intrusion of mercury for a pore size under 1 µm is relevant for the intraparticulate pore space (Figure 9).

#### 3.3.9. Flow Properties

Numerous operations during the manufacturing of dosage forms require the handling and processing of powders. These operations include blending, transfer, storage and, for the final dosage, form processing, tableting and capsule filling. In order for these processes to be conducted in a predictable and reproducible way, powders must exhibit acceptable flow properties [69]. It has been proved that poor flow properties negatively affect tablet and capsule weight uniformity [70,71]. Solidified LBDDS systems are prone to cohesiveness because of lipid components being inherently sticky; therefore, flow properties of spray dried powders were measured and analysed.

##### 3.3.9.1. Hausner Ratio

**Figure 10:**
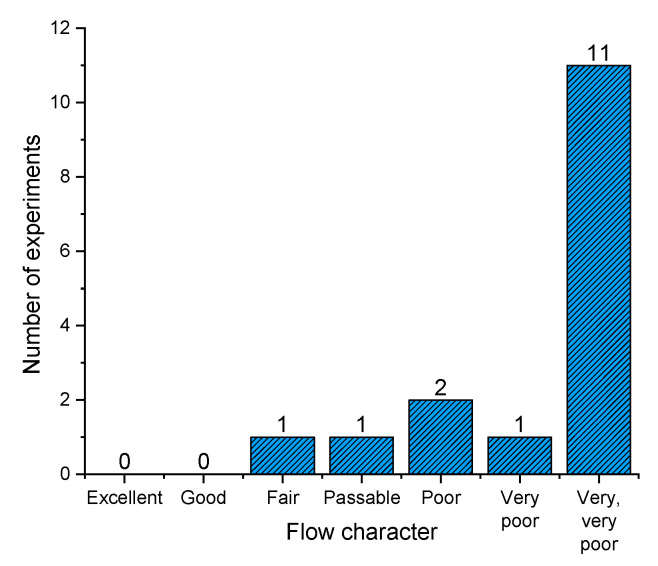
Dry emulsion spray dried powders according to flow character derived from HR values.

In order to verify various parameters obtained during rotating drum experiments, Hausner ratio, as a classic, compendium, easy-to-obtain index was also assessed for powder samples. Hausner ratio is based on the difference between the tapped and the bulk density, and although it has a less theoretic base, it has been used for many decades to describe powder flow [72]. One can see from Figure 10 that the majority of obtained powders rank in the very, very poor Hausner ratio flow character. However, we can see that two powders have fair (F14) and passable (F13) flow characteristics, which could be used for further processing, without expecting problems associated with flowability. It has been demonstrated that a Hausner ratio of 1.25 (and above) is the value where flowability, and hence tablet weight variation, for example, becomes more pronounced [70,73].

**Figure 11:**
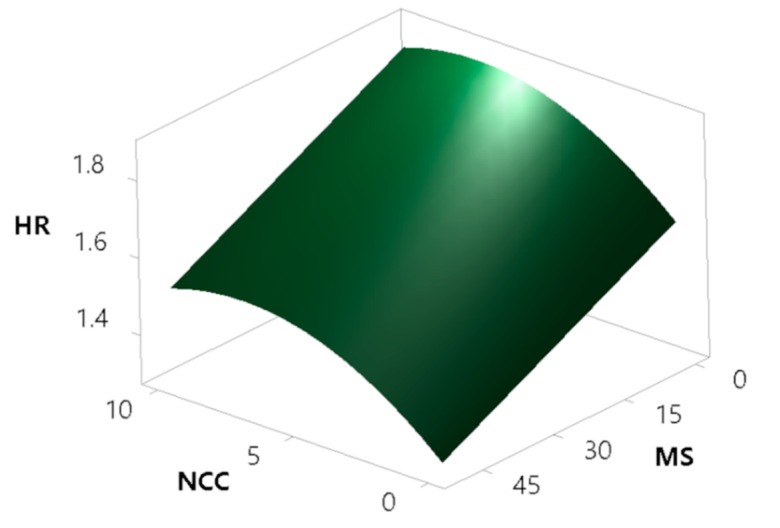
Surface plot showing the effect of the concentrations of MS and NCC on Hausner ratio (HR).

The modelling of HR results has been performed, yielding a model with high coefficients of determination (R^2^ = 0.8561, R^2^(adj) = 0.8229, R^2^(pred) = 0.7429). The modelling surprisingly showed that the concentration of the oil phase does not have a significant effect on powder flowability. This can be explained by the fact that MS absorbs at least part of the oil phase in the pores, and the addition of NCC and the soluble matrix materials covers the majority of the oil remaining on the surface, diminishing the amount of free oil exposed on the particle surface, which could otherwise act as a sticky agent impairing flowability. According to the model (Figure 11), NCC has a deteriorating effect on powder flow properties, which is expected to an extent, as rod-shaped NCC crystallites on the surface of MS intercalate MS particles and thus contribute to the worsening of the powder flow. On the other side, powder flow is improved by the addition of MS. The reason behind this is due to two different factors: MS increases the average particle size of the product and also exhibits a high envelope particle density, which directly translates to improved flow properties. MS particles also absorb oil into the pores, thus reducing the quantity of oil on the surface.

##### 3.3.9.2. Avalanche Testing

HR is an easy to measure and repeatable index; however, it has some drawbacks. HR sometimes does not correlate well with other flow property estimators, and sometimes different materials with the same HR value will behave differently in the real environment of material processing. Due to this, additional tests have been developed, with the rotating drum technique being one of them. The main advantage of the rotating drum technique is that it is a dynamic method, meaning that it measures the sample flow properties with powder in motion, which, for certain processes, is a better representation of the actual powder flow behaviour [74].

The avalanche angle median is the median of all the powder bed angle measurements, which are taken at the sample position prior to the start of the avalanche. The avalanche median for the samples of the 17 experiments ranged from 60.0° to 109.4° for experiment F14 and experiment F16, respectively. Modelling of the avalanche angle median results has been performed. The model with high coefficients of determination (R^2^ = 0.9443, R^2^(adj) = 0.9257, R^2^(pred) = 0.8598) is described by Equation (11):

(11) $$\mathrm{Avalanche}\;\mathrm{angle}\;\mathrm{median}=98.06+448\times X2+2.4\times X3-3187\times X2^{2}-150.7\times X3^{2}$$

In this case, the results perfectly align with the HR results, confirming the positive effect of the MS and the negative effect of NCC. In a study employing the same instrument, Rao Nalluri et al. have shown that API-excipient mixtures having avalanche angles bellow cca. 64° led to a relative standard deviation of 2% in capsule weight during capsule filling, using an automated pilot-scale machine [75]. Looking at these results, we can speculate that F13 and F14 would be suitable for the process of capsule filling, as both products demonstrated avalanche angles below 64°.

**Figure 12:**
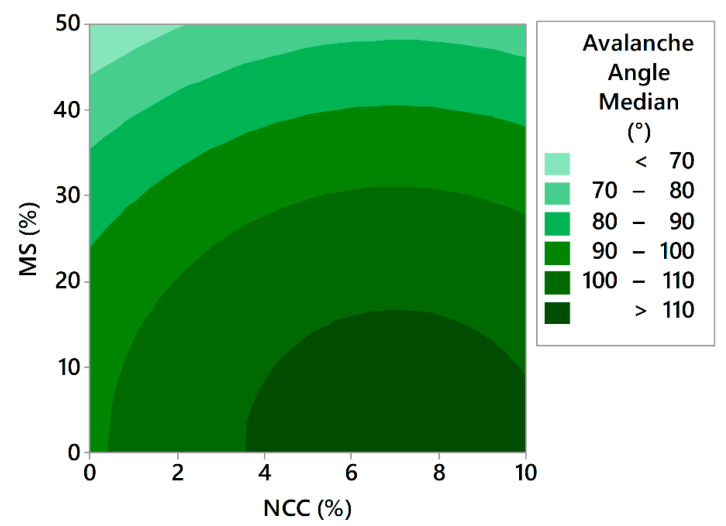
Effect of the concentrations of both insoluble matrix formers on avalanche angle median.

**Figure 13:**
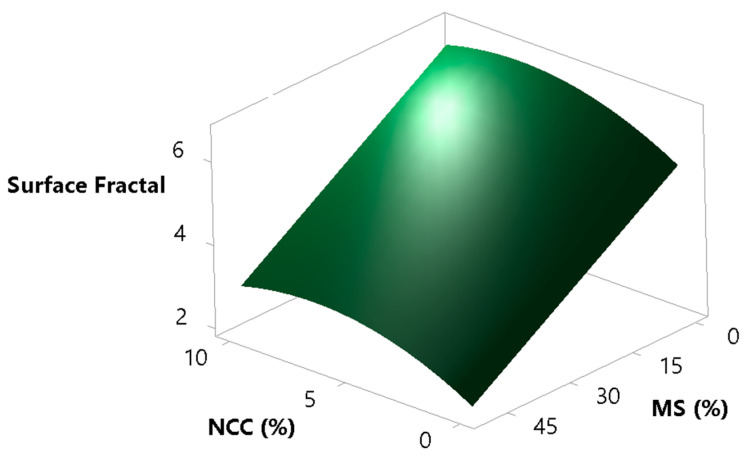
Surface plot showing the effects of the concentrations of MS and NCC on the sample surface fractal.

Surface fractal is the fractal dimension of the surface of the powder bed and gives the information on how rough the surface of the powder bed is during its movement. The measurement is taken after each avalanche and is dependent on the ability of the powder to reorganise itself to a smooth surface. This is an indirect measure of inter-particle forces and is of great importance, where an even volumetric filling of powders is essential, as in the case of die filling and capsule filling. For a smooth powder bed surface, we would have a surface fractal value close to the value of unity, and for a rough powder bed surface, we would have a number greater than one [76]. The goal was to verify whether the surface fractal results correlate well with the results of the avalanche angle and the HR testing. By looking at Figure 11 and Figure 12, one can clearly see that the model of the surface fractal (Figure 13) aligns very well in terms of shape with the HR model and avalanche angle median model. The surface fractal model also had high coefficients of determination (R^2^ = 0.9190, R^2^(adj) = 0.9003, R^2^(pred) = 0.8575), which confirms the reliability and conclusions drawn from the models.

#### 3.3.10. Dissolution

**Figure 14:**
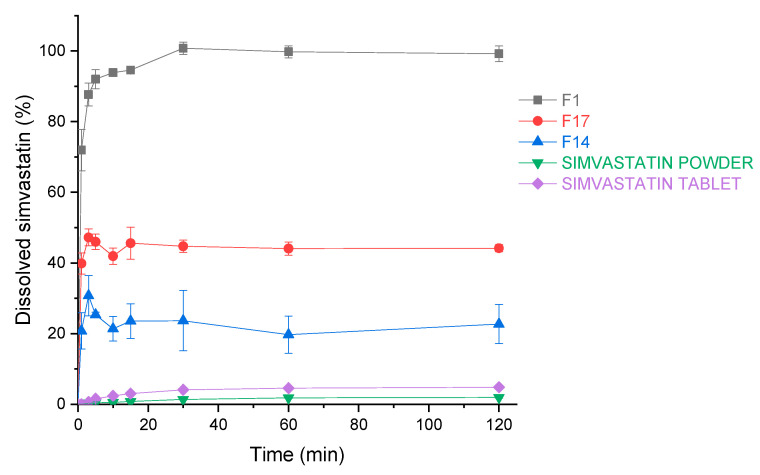
The dissolution profiles of simvastatin for simvastatin powder, simvastatin tablets and experiments F1, F14 and F17. Each data point is expressed as the mean percentage ± SD of three determinations.

Dissolution constitutes an essential step in drug absorption from orally ingested pharmaceutical dosage forms. As the scope was not only to evaluate the total percentage released from the dosage form (Section 3.3.4 Amount of released drug), but also the rate of the release, we carried out a dissolution study and compared three spray drying products with pure simvastatin powder and a generic simvastatin tablet. The three formulations tested were: F1 without insoluble matrix formers, F17 with both insoluble matrix formers at the highest concentration and F14 where only MS at the highest concentration was present (without NCC). Tested formulations outperform the pure drug and the generic tablet containing simvastatin (Figure 14). The increase in dissolution after 120 min (time relevant for drug absorption in GIT) is 51.3, 22.8 and 11.7 times higher for experiments F1, F17 and F14, respectively, compared to the pure crystalline simvastatin, as can be seen from Figure 14. By comparing samples of the three selected spray drying experiments, one can observe that only F1 achieves a fast and complete release after 120 min. On the other hand, F14 exhibits only 22.7% of drug release after 2 h, which confirms our conclusions from the release tests, showing an inhibited release of simvastatin due to MS entrapment of the oil phase. However, the addition of 10% of NCC (F17) enhances the percentage of the released drug after 120 min by 21.5%, thus demonstrating its ability to alleviate desorption of the oil phase and hence, simvastatin from MS.

#### 3.3.11. Concurrent Improvement of Flow Properties and Dissolution

By combining the DoE results from the flowability and release studies, it can be seen that one cannot improve one characteristic without negatively impacting the other. If a high MS concentration is used, oil remains entrapped within MS particles during dissolution. On the other hand, if NCC is used, the release of oil is improved; however, flow properties are being hindered by the rod-shaped particles of NCC deposited on the surface of MS particles.

After the DoE study was completed, an additional small number of experiments were performed, as a proof of concept study, by employing the same formulation components, but using an alternative method of dry emulsion powder preparation. In this case, a suspension of MS in water was first prepared, and then NCC was added to the suspension and then spray dried. The rationale behind this approach was that we would firstly cover the inside of MS pores before adding the API dissolved in the oil—surfactant mixture. After the spray drying, the powder was collected and then finally mixed in the mortar by the dropwise addition of the oil–surfactant mixture in the ratios per dry powder, as shown in Table 2. The amount of simvastatin in the oil—surfactant mixture was kept the same as in the main study, i.e., 70 mg/g. With this approach, we wanted to maximise the release of the drug from the dry emulsion, specifically from MS. Three different formulations were prepared and then tested for the release of the drug, as shown in Table 2. Obtained results demonstrate that the higher the ratio of NCC to MS, the higher the amount of the released drug. However, by increasing the NCC to MS ratio, the total capacity of the matrices powder to accept the oil phase decreases. Only half of the oil phase could be added in case of formulation S3, when compared to formulations S1 and S2. If higher amounts of the oil phase are used, a sticky mass is formed.

The presented preliminary proof of concept study demonstrates that by combining a preparation step, which involves spray drying NCC and MS alone and the mixing of the produced powder with the oil—surfactant mixture, one can achieve a high drug release from the MS system. Although both steps could be performed with processing equipment (spray dryer and high shear mixer), the described procedure is a two-step process and, consequently, more time consuming, thus probably less appealing to the industry.

## 4. Conclusions

The presented study discloses the potential of employing a new combination of two rather novel materials for the production of dry emulsion systems using the spray drying process. The DoE approach used in this study allowed for thorough study of the effects each individual formulation parameter and their interactions have on selected product attributes.

In total, 17 emulsion/suspension preparation and spray drying experiments were performed based on the response surface design. A number of relevant results stood out after the DoE analysis was performed. Soluble matrix formers (mannitol and HPMC) are more efficient in encapsulating oil droplets compared to the MS and NCC system. NCC did not have a significant effect on encapsulation efficiency in the concentration range used. Based on all three evaluation metrics, the employed MS concentration strongly improved the flow properties, as a consequence of particle size increase; however, MS significantly deteriorated simvastatin release by entrapping it in the pores, not allowing its release. On the other side, the addition of NCC impaired the flow properties, most probably due to the rod-shaped particles; however, it improved the simvastatin release. The latter is most probably a consequence of NCC covering a portion of the inner part of the pores, enabling easier oil desorption. With regards to the ability of the dry emulsion system to reconstitute, the MS phase improved the redispersibility of the studied dry emulsion system; however, NCC showed a negative effect.

In conclusion, we note that MS and NCC are materials with a low toxicity and great potential for designing dry emulsion systems via the spray drying process. Each component has its own advantages and disadvantages regarding product characteristics, and concurrent optimisation of both flow properties and drug release is hindered, as by improving one product characteristic, we impair the other. The presented preliminary proof of the concept study has however demonstrated that by modifying the procedure, one could find a solution to the challenges that stem from the DoE study. Therefore, new combinations with other materials and/or modifications of the process should be explored in order to further improve the proposed drug delivery system.

## Figures and Tables

**Table 1:** Surface response experimental design, with three variables: oil phase (1-oleoyl-rac-glycerol with Miglyol^®^ 812—9:1, surfactant—Tween^®^ 20 * and simvastatin), NCC and MS (F1–F17). The oil phase percentage ranged from 27% to 40%, the NCC percentage ranged from 0% to 10% and the MS percentage ranged from 0% to 50%.

Experiment	Oil Phase (%) X1	NCC (%) X2	MS (%) X3	Soluble Matrix Formers (%)
F1	27	0	0	73
F2	40	5	25	30
F3	40	10	0	50
F4	33.5	10	25	31.5
F5	33.5	5	25	36.5
F6	33.5	0	25	41.5
F7	40	0	0	60
F8	33.5	5	50	11.5
F9	33.5	5	0	61.5
F10	33.5	5	25	36.5
F11	27	5	25	43
F12	27	10	50	13
F13	40	0	50	10
F14	27	0	50	23
F15	33.5	5	25	36.5
F16	27	10	0	63
F17	40	10	50	0

* Surfactant concentration was kept at 0.5% for all experiments.

**Table 2:** Ratio between MS, NCC and the oil—surfactant mixture of three prepared formulations and the percent of released drug from each formulation, determined according to the method described under Section 2.2.5.2. Amount of released drug.

Formulation	MS	NCC	Oil Phase	Released Drug (%)
S1	2	1	0.68	46.4%
S2	1	1	0.68	72.3%
S3	1	2	0.34	84.5%

## Data Availability

The data presented in this study are contained within the article.
